# Biological properties of *Moringa oleifera*: A systematic review of the last decade

**DOI:** 10.12688/f1000research.157194.2

**Published:** 2025-01-30

**Authors:** Javier Andrés Soto, Andrea Catalina Gómez, Maryeli Vásquez, Andrea Natalia Barreto, Karen Shirley Molina, C. A. Zuniga-Gonzalez

**Affiliations:** 1 Facultad de Ciencias Médicas y de la Salud, Instituto de Investigación Masira, University of Santander, Bucaramanga, Santander, 540003, Colombia; 2 Area of knowledge of Agrarian and Veterinary Sciences Research Centre, Bioeconomy and Climate Change Unit Research, National Autonomous University of Nicaragua, Leon, Leon, Leon, 21000, Nicaragua

**Keywords:** Biological activity, cancer, chronic diseases, infectious diseases, Moringa oleifera, phytochemical components.

## Abstract

**Background:**

The growing incidence of chronic diseases such as cancer and the emergence of drug-resistant microorganisms constitute one of the greatest health challenges of the 21st century. Therefore, it is critical to search for new therapeutic alternatives. *Moringa oleifera* is a plant well known for the properties of its phytocomponents and its role has been analyzed in a variety of fields, from medicine to biotechnology.

**Methods:**

In this work, the biological activity of *Moringa oleifera* in human health was explored through a review of 129 original articles published between 2010 and 2021 related to antitumor activity and its potential uses against chronic and infectious diseases.

**Results:**

*Moringa oleifera* extracts showed antioxidant, hypoglycemic, antihypertensive and cytoprotective properties at neuronal, hepatic, renal and cardiac levels. Besides, cytotoxic effects, apoptotic and antiploriferative activity against several cancer cell lines has been demonstrated. On the other hand, the antimicrobial potential of *M. oleifera* was also evidenced, especially against multidrug-resistant strains.

**Conclusions:**

Hence, it is supported that there is a wide range of clinical entities in which *Moringa oleifera* exhibits significant biological activity that could contribute to counteracting metabolic, infectious and chronic diseases in a similar or improved way to the drugs traditionally used.

## 1. Introduction

Natural resources, such as plants, have been used since ancient times to treat various diseases with positive results. Recently, the use of medicinal plants has gained significant interest in low-and middle-income countries. Among them, *Moringa oleifera* has emerged as a plant with immense potential for health and industry due to the beneficial properties of its components. ^1^

*Moringa oleifera* (MO) is a globally distributed Indian native tree whose various parts, including bark, leaves, roots, seeds, pods, fruits, and flowers, are consumed as therapeutic alternative to treat a wide range of ailments. This plant, particularly its leaves, ^2^ is highly nutritious, containing significant amounts of vitamins (A, C and E). Vitamin A plays a key role in vision, immunity, cell growth, differentiation and reproduction while vitamins C and E protect against free radicals, acting as potentantioxidants. The leaves are also rich in essential amino acids and minerals such as iron, phosphorus, potassium and calcium. Calcium is particularly crucial for blood coagulation, maintaining the nervous system, and supporting bone and tooth health, as well as helping delay the onset of osteoporosis. Furthermore, MO contains a variety of bioactive compounds, including terpenoids, anthraquinones and glycosides. ^1– 3^

In addition to its nutritional value, *Moringa oleifera* has a low fat content (4.03-9.51%) with polyunsaturated fatty acids predominating over saturated fats. This suggests a potential link to the prevention of coronary heart disease. ^3^ Moreover, MO is rich in fiber, contributing to the detoxification of the gastrointestinal tract. The plant’s presence of isothiocyanates also grants it antibacterial properties. ^4^

Several studies have highlighted MO as a valuable plant providing both nutritional and medicinal benefits. However, the majority of studies have focused on *in vitro* analyses, with limited *in vivo* evidence, partly due to concerns about its cytotoxicity. While *Moringa oleifera* shows significant therapeutic potential, a key gap remains in understanding its *in vivo* effects, particularly concerning the variability of its efficacy and potential toxicological risks. This review aims to consolidate the evidence on newly identified mechanisms of action, particularly its role in modulating specific molecular pathways related to cancer progression and bacterial resistance. ^1^

Recent research has emphasized the leaf as the part of the plant with the highest biological potential, containing an array of vitamins and amino acids. Additionally, it demonstrates significant water purification capacity and exceptional nutritional value. ^4^ Despite these benefits, studies, such as one by Sultana (2020), report discrepancies regarding the protein content in the leaves, suggesting that environmental factors may influence its composition. ^3^

This review specifically examines the dual therapeutic potential of *Moringa oleifera* in addressing two critical global health challenges: cancer and antimicrobial resistance. By integrating findings from diverse fields such as medicine, biotechnology, and phytotherapy, this work offers a broader perspective compared to prior reviews that often focus on single disciplines. The data analysis presented here reflects the most relevant findings and explores the plant’s potential for overcoming limitations of conventional therapies, such as side effects, low specificity, and limited efficacy.

### 1.1 *Moringa oleifera*

*Moringa oleifera* is a tree belonging to the *Moringaceae* family, native to the sub-Himalayan area of India, Pakistan, Bangladesh and Afghanistan whose genus has 13 species distributed from tropical to subtropical regions. ^5^ MO is a plant widely studied worldwide for its components since these confer it particularities of interest in areas such as health, aesthetics, and nutrition, among others.

The most studied part is its leaves since these have been attributed different biological properties including antimicrobial, hypotensive, antiulcer, hypocholesterolemic, antispasmodic, antioxidant, anti-inflammatory and antitumor agent potential. ^6^ In relation to its chemopreventive role, Sreelatha *et al.* ^7^ evaluated the antitumor effect of *Moringa oleifera* leaf alcoholic extract (rich in phenolic compounds, such as quercetin and kaempferol) on glandular cervical cancer cells, revealing a potential in the inhibition of cell viability through the induction of apoptosis and oxidative stress through the generation of reactive oxygen species (ROS) and an antiproliferative effect, thus revealing a promising outlook as an antitumor alternative. ^7^

MO is one of the most cultivated and important medicinal plants in India and is considered as a staple food in various parts of the world since its valuable nutritional potential ^4^ due to the high content of minerals and vitamins such as iron, potassium, copper, zinc, magnesium, manganese, vitamin C, vitamin E, beta-carotene, among others. Different studies have been described associated with the leaf, seed, fruit, bark and flower of *Moringa oleifera* in several types of cancer, which have revealed the biological antitumor activity both *in vitro* and *in vivo.* One of these works was performed by Hussein *et al.* ^8^ in which they determined the components of *Moringa oleifera* seed essential oil *in vitro* that act as antioxidants, and showed the cytotoxic potential over tumor cell lines like human breast adenocarcinoma (MCF-7), colon carcinoma (HCT-116), human hepatocarcinoma (HepG2) and HELA cells (8).

### 1.2 Phytochemical features

**Figure 1.**
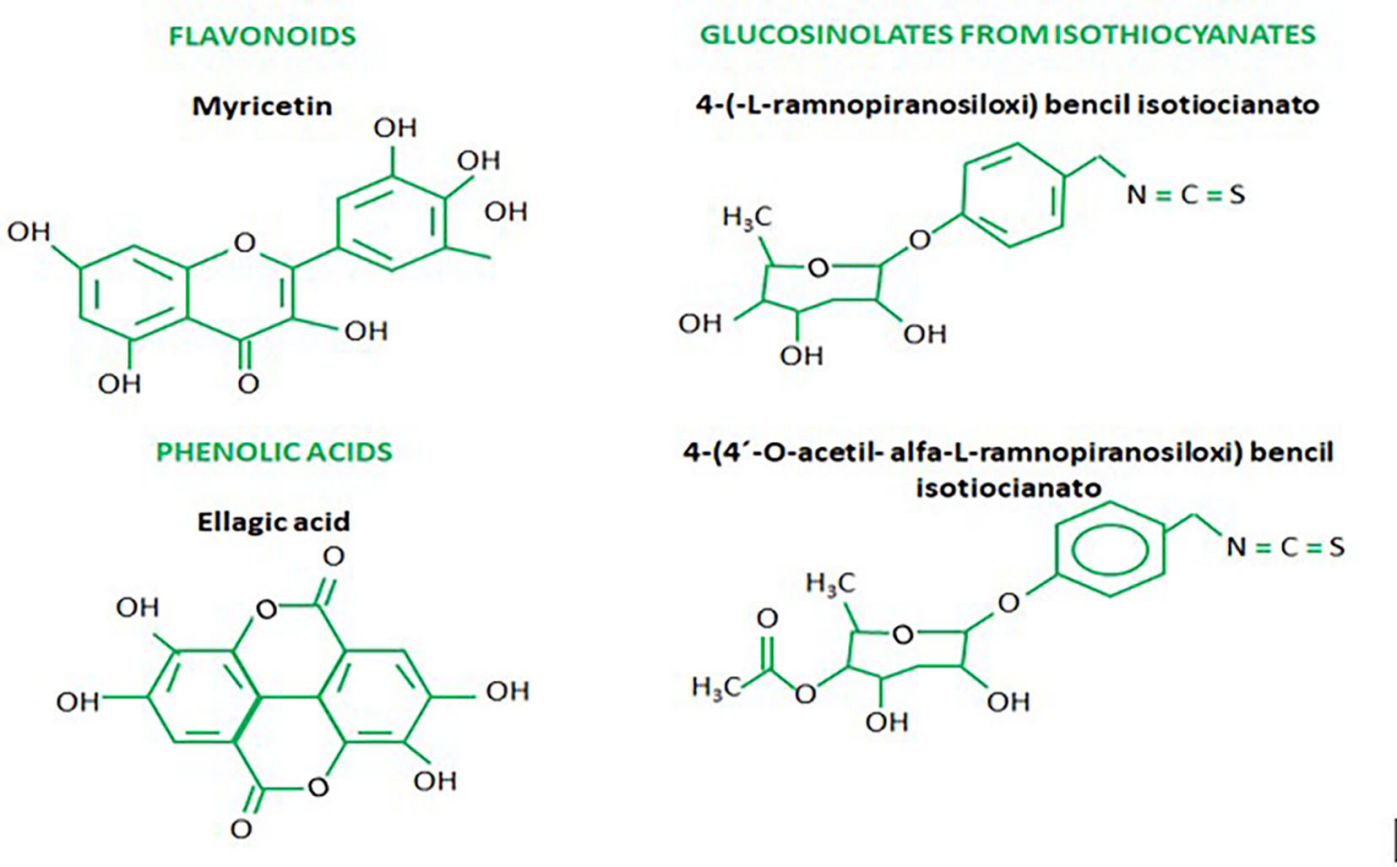
Chemical structures of the most significant secondary metabolites of *Moringa oleifera*.

This plant has been considered of great curative relevance in relation to the presence of significant phytochemicals ^9^ also known as secondary metabolites such as polyphenols, tannins, sterols, terpenoids, flavonoids, saponins, anthraquinones, alkaloids and some reducing sugars mentioned along with anticancer elements, including glucosinolates, isothiocyanates, glycoside compounds and glycerol-1-9-octadecanoate. ^10^ Phenolic acid derivatives are elements with similarity to flavonoids, which have been related to plant protection against UV rays and also with other defensive mechanisms. One type of phenolic acid, chlorogenic acids, are known for protecting against diseases caused by oxidative stress. ^11^ Some of the main flavonoids found by Sultana and Anwar (2008) present in *Moringa oleifera* leaves are myricycin, quercetin and kaempferol, at concentrations of 5.8, 0.207 and 7.57 mg/g, respectively. ^12^ Moreover, Vergara-Jiménez *et al.* ^13^ point out that quercetin is found in MO dried leaves in concentrations of 100 mg/100 g. This phytochemical acts as a strong antioxidant and exhibits hypolipidemic, hypotensive and antidiabetic potential, which has been demonstrated *in vivo* with rats suffering from metabolic syndrome and rabbits with hyperlipidemia ( Figure 1). ^13^

Concerning the biological activity of MO against microorganisms, Padla *et al.* ^14^ highlight that isothiocyanates isolated from *Moringa oleifera* seeds showed inhibitory activity at the lowest concentration of 1 mg/ml towards all Gram-positive bacteria tested ( *Staphylococcus aureus*, *Staphylococcus epidermidis* and *Bacillus subtilis*) and against the dermatophyte fungi *Epidermophyton floccosum* and *Trichophyton rubrum*. ^14^

### 1.3 Traditional uses

Several elements of this plant have been used traditionally in India as a preventive and treatment approach for pathologies such as asthma, gout, rheumatism, and infections. ^15^ Same scenario occurs in Africa where this MO is used to combat arthritis, joint pain, headaches, abdominal pain, otitis, and dental pain and it has also been used as a cardiac and circulatory stimulant, in individuals with asthenia, intestinal parasites, febrile periods, renal and hepatic complications, epilepsy, anemia, ulcers, delirium, and trauma caused by snakes.

*Moringa oleifera* has been used to treat respiratory diseases such as influenza and asthma, and even SARS-CoV-2, which caused the last pandemic, ^16^ gastritis, headaches and flatulence ^17^; thus demonstrating its wide range of uses for a variety of clinical entities. It should be remembered that millions of people in the world do not have access to modern medicine, having to resort to the alternatives provided by ancestral medicine. ^18^

### 1.4 Antioxidant effect

The relevance of the antioxidant activities of components that make up the *Moringa oleifera* plant, such as polyphenols, alkaloids, saponins, carotene, minerals, amino acids, and sterols, has been demonstrated. Such antioxidant features has been identified through several approaches, including colorimetric methods such as DPPHH (2,2-diphenyl-1-picrylhydrazyl), ABTS [2,2′-azino-bis-(3-ethylbenzothiazoline-6-sulfonic acid)], LPO (lipid peroxidation), FRAP (ferric reducing antioxidant power), among others, in order to obtain evidence related to the redox potential of the plant. ^19^

Polyphenols are phytochemicals found in large quantities especially in *Moringa oleifera* and are considered natural antioxidants. These are plant compounds that are attributed with the property of indirectly reducing oxidative damage in tissues by strengthening cells or eliminating free radicals. ^20^ It has been demonstrated that consumption of polyphenols protects against chronic pathologies related to oxidative stress, including cardiovascular diseases and cancer. ^13^ The presence of polyphenols in high concentrations in this plant leaves opens the door for their use in the aforementioned diseases. It is worth noting that to achieve the greater potential of antioxidants against damage caused by free radicals, the synergistic work of several antioxidants is necessary. A combination of effective antioxidants n the leaves of *Moringa oleifera* has been found ^21^ among then, polyphenols, mostly flavonoids (myricetin, quercetin, and kaempferol), together with phenolic acids (chlorogenic acid, caffeic acid, and gallic acid). ^22^

*Moringa oleifera* extracts exhibit strong antioxidant activity and radical scavenging *in vivo*, protecting animals against oxidative stress-induced diseases. Consumption of leaf and seed extracts has shown significant therapeutic properties against diabetes and hypertension due to the phyto-components, exhibiting mitochondrial redox potential, increasing heme oxygenase-1 and reducing reactive oxygen species (ROS) induction as well as lipid peroxidation, thus conferring protective effects against negative effects associated with obesity. ^23, 24^ It is even suggested that its antioxidant capacity is so promising that the role of *Moringa oleífera* derivatives should be explored in the treatment of wastewater and as a future source of important nutraceuticals. ^21^

### 1.5 Anticancer activity

The potential of *Moringa oleifera* as an anticancer agent has been elucidated through the role of ethanolic extracts obtained from the leaves and bark which have effectively inhibited the growth of cancer cells in breast, pancreas, and colorectal tissue. It is believed that the plant’s antitumor capacity is due to the presence of isothiocyanates, glucosinolates, glycosylated compounds, and glycerol 1-9-octadecanoate. ^25^ It has also been proved that *Moringa oleifera* pods act as a chemo preventive agents *in vivo*. This protective effect has also been identified by Shaban *et al.,* ^26^ who observed an in-crease in antioxidant levels and a reduction in the formation of free radicals against several cancer cell lines (lung, liver, colon, and neuroblastoma) treated with the plant. ^26^

Regarding the extract of *Moringa oleifera* seed, this has shown specific growth inhibitory effects reflected in a 95% inhibitory activity on neuroblastoma cell line. ^27^ In some cases, tumors treated with methanol extracts from fruits and leaves have grown slowly, indicating effective cell degradation. Likewise, it has been found that the extract is more effective in terms of volume, since at a concentration of 500 mg/kg, the doubling time and growth delay indicate tumor-suppressing properties. ^27^

## 2. Methods

This compilation is of a qualitative, descriptive and documentary nature and its development required the collection of original scientific articles obtained from databases such as PubMed, ScienceDirect, EBSCO Host, Scopus, and Google Scholar. The number of records identified through the database search was 2700 publications initially. Subsequently, these raw results were processed using filters such as keywords including *Moringa oleifera*, chronic diseases, infectious diseases, biological activity, human cancer, antitumor biological activity, *in vivo* and *in vitro* activity and anticancer properties, aiming to identify and select relevant information for the analysis of biological activity against human diseases, thus reducing the number of potential publications to 410.

From the articles found in the above-mentioned databases, an exclusion and inclusion process was carried out. Once the data was obtained, this information was housed in a categorization matrix that specified different items such as access link, title, authors, journal, year, methodology, results, conclusions, and discussions aiming to synthesize the information and making it more accessible for a selective and exclusive analysis. This screening and analysis process allowed to obtain a total of 112 definitive publications. It is important to highlight that not only the articles described in the results section were used, this means that there is a greater number of articles mentioned and analyzed since other bibliographic sources were added in the rest of the body of the document.

A systematic literature review was conducted using academic databases such as PubMed, Scopus, and Google Scholar. Original articles published between 2010 and 2021 that investigated the biological activity of *Moringa oleifera* in relation to its antitumor activity and potential uses against chronic and infectious diseases were included.

### 2.1 Inclusion and exclusion criteria

Inclusion criteria included studies that:

a) Addressed the biological activity of *Moringa oleifera.*
b) Presented empirical results regarding its antitumor properties or effects on chronic and infectious diseases.
c) Were original articles peer-reviewed.
d) Exclusion criteria comprised reviews, editorials, and articles not published in English or Spanish.

### 2.2 Review process

A search was performed using keywords such as “ *Moringa oleifera*,” “antitumor,” “chronic diseases,” and “infectious diseases.” Following the initial search, titles and abstracts were screened to filter relevant articles. A full reading of the selected articles was then conducted to extract data on biological activity and proposed mechanisms.

### 2.3 Data analysis

The findings from the selected articles were synthesized, highlighting the most relevant biological properties and methodologies employed in each study. A descriptive approach was used to summarize results and discuss the implications of the findings for human health.

## 3. Results and discussion

### 3.1 Chronic, infectious diseases and cancer

Chronic diseases are characterized by a long course and slow progression, and include cardiovascular diseases, cancer, chronic kidney and liver diseases, rheumatic disease, and diabetes. According to the World Health Organization (WHO), chronic diseases are the leading cause of mortality worldwide, causing approximately 41 million deaths each year, equivalent to 71% of deaths globally. ^28^ These pathologies can be prevented by modifying common behavioral risk factors, such as smoking, alcohol consumption, unhealthy diet, and physical inactivity, which contribute to the development of metabolic changes that usually manifest themselves as hypertension, obesity, hyperglycemia, and hyperlipidemia. ^29^

Specific genetic events and external agents such as physical carcinogens such as ultraviolet (UV) and ionizing radiation, chemical carcinogens such as asbestos, aflatoxins, arsenic, and biological carcinogens (e.g. *Helicobacter pylori*, viruses, and parasites) increase disease morbidity. ^30^ However, cardiovascular diseases are positioned before cancer, as they are the leading cause of death among chronic diseases (approximately 18 million deaths per year), followed by cancer (9 million deaths per year) and diabetes (1.6 million deaths per year). ^28^ These three pathologies are responsible for more than 80% of premature deaths, that is, fatal events occurring between the ages of 30-69 years. ^29^

Concerning infectious diseases, which are basically foodborne illnesses (FBIs), sexually transmitted infections (STIs) such as HIV/AIDS, acute respiratory infections (ARIs) including the common flu and the current novel coronavirus (COVID-19), and other parasitic and bacterial diseases; a global increase in drug resistance rates has been identified, indicating a loss of effectiveness in usual treatments. Regarding antiviral resistance, there is an increasing concern, mainly for immunocompromised patients, as well as for the emergence of drug-resistant parasites, which constitute one of the greatest threats to malaria control. In the case of drug-resistant fungal infections, the therapeutic situation is already difficult se because patients currently experience treatment problems, such as toxicity, especially in individuals with other underlying infections. ^31^ Some infections can be prevented by vaccination; however, the increasing resistance to antimicrobials (antibiotic, antiviral, antifungal, and antiparasitic) due to their inappropriate and excessive use has led to some of these diseases becoming a global public health problem owing to an increase in morbidity and mortality rates, long hospital stays, and high healthcare costs. ^31^

Therefore, considering what has already been described, a viable therapeutic alternative is found in natural and complementary medicine based on the use of plant material, its extracts, and derivatives. In recent decades, medicinal plants have become a trend in developing countries, with *Moringa oleifera* as a promising plant because several studies have demonstrated its therapeutic and pharmacological properties, including neuroprotective, antimicrobial, antiasthmatic, antimalarial, cardioprotective, antidiabetic, **antiobesity**, hepatoprotective, and anticancer effects. ^1^

Chronic diseases have had a significant impact worldwide due to their high mortality rates and the fact that their related therapies often have side effects on patients. Based on this, there is a need to explore other therapeutic alternatives, such as natural treatments, including the use of *Moringa oleifera*, which has shown positive effects regarding the potential treatment of such diseases over time, as revealed in this study. In the following sections, several *in vitro* and *in vivo* studies conducted between 2010 and 2021 on the use of *Moringa oleifera* in the aforementioned clinical events are described.

### 3.2 Biological activity of *Moringa oleifera* in chronic diseases

Cardiovascular diseases are chronic diseases and are of great interest because they rank first as the cause of death worldwide. However, in chronic diseases, another clinical entity attracts the attention of the medical community because of the rapid increase in incidence rates and high associated morbidity. According to data from the Diabetes Atlas, 463 million adults currently live with this disease, and the global projection of cases for 2025 is 438 million, but this prediction has already been exceeded by 25 million cases. ^32^ Making the scenario more complex, this pathology is not diagnosed or is misdiagnosed in low-income countries, increasing the risk of serious complications and mortality. Additionally, the lack of availability of diabetes medications is a challenge. The projections for this disease are not only gloomy regarding the well-being of individuals but also in terms of fiscal detriment to global health systems, as expenses related to the disease are estimated to reach over one billion dollars by 2045. ^33^

**3.2.1 Therapeutic properties for chronic diseases**

In India, the native country of the plant, Aju *et al.* ^34^ evaluated the protective role of the methanolic extract of *Moringa oleifera* leaves (MOME) in the hearts of diabetic rats and demonstrated that treatment with MOME at a dose of 300 mg/kg/day significantly reduced hyperglycemia and oxidative stress in the hearts of the animals. ^34^ The potential antidiabetic and antioxidant properties of MOME were attributed to the presence of various biologically active compounds, such as heptadecanoic acid, hexadecanoic acid, DL-alpha-tocopherol, 11-14-17-eicosatrienoic acid, and 9-12-15-octadecatrienal, which have been previously associated with the antiarthritic and anticoronary activities of the plant. ^34^

In line with the antidiabetic study, Khan *et al.* ^35^ used the aqueous extract of *Moringa oleifera* leaf (AEMOL) at a dose of 100 mg/kg in streptozotocin-induced diabetic rats to determine the hypoglycemic potential to assess the enzymatic activity, seeking to identify the antioxidant capacity due to the presence of compounds such as phenols. ^35^ However, the evaluation of inhibitory activities and potential radical scavengers of two promising plants ( *M. oleifera* and *T. occidentalis*) on the enzymes highlighted in diabetes mellitus revealed enzyme inhibition, ^36^ with *Moringa oleifera* being the major contributor to this event in a dose-dependent manner. Another relevant finding of this study was related to the identification of a greater number of phytoconstituents in *Moringa oleifera* than in *Telfairia occidentalis.* ^36^

Concerning the antidiabetic role of *Moringa oleifera* leaf powder, the chemical composition of this substance has been evaluated with regard to the inhibition of α-amylase enzyme, testing the inclusion of 20 g of *Moringa oleifera* leaf powder in natural foods and studying in diabetic and healthy patients. From these analyses, it was found that *Moringa oleifera* leaf powder contains a high amount of protein, fiber, and trace elements, in addition to known polyphenols. ^37^

Approaches focused on demonstrating an association between the potential of leaf extracts and therapy for vascular diseases have evaluated the antihypertensive mechanisms of the aqueous extract of *Moringa oleifera* leaves (AEMOL). Aekthammarat *et al.* ^38^ concluded that AEMOL induces the synthesis of Nitric Oxide (NO) in endothelial cells, thus reducing peripheral vascular tone and blood pressure (BP), a dependent event on soluble guanylate cyclase (sGC). This induces vasorelaxation through the activation of the eNOS-NO-sGC pathway, allowing us to understand the pharmacological properties of AEMOL against arterial hypertension. ^38^

The seeds of *Moringa oleifera* seeds have also shown potential for exhibiting antidiabetic effects. Al-Malki and Rabey ^39^ tested low doses of *Moringa oleifera* seed powder (50 and 100 mg/kg) in rats with induced diabetes. Using various methodological approaches, such as biochemical, immunological, physiological, and histological analyses they observed that low doses decreased lipid peroxidation to a greater extent than the 100 mg/kg concentrations. They also found that antioxidant enzyme levels increased in the treated groups. Specifically, regarding glycosylated hemoglobin, rats treated with *M. oleifera* showed significantly reduced values, although the most drastic change was associated with the dose of 100 mg/kg. ^39^

The methanolic extract of *Moringa oleifera* seeds (MEMOS) has been evaluated for its ability to alter serum lipid profiles. An example of this is the assessment carried out by Ajayi *et al.,* ^40^ where weight gain was observed in untreated animals compared to those treated with this extract. It was also noted that there was a decrease in total cholesterol (<0.05) in experimental animals administered MEMOS at 100 mg/kg, but this effect was not significant at 200 mg/kg. Regarding high-density lipoprotein (HDL), it was found that in groups treated with both concentrations of MEMOS, this parameter increased, but this increase was more significant at 200 mg/kg. ^40^

Table 1 shows some *in vivo* and *in vitro* concerning the potential of extracts obtained from different parts of the plant to counteract some chronic diseases.

**Table 1.** Potential uses of *Moringa oleifera* in chronic diseases.

Plant part	Extract fraction	Disease	Effects	Mechanisms	References
Leaf	Methanolic, aqueous, powder, ethyl acetate	Diabetes mellitus	Hypoglycemic	Inhibition of α-amylase and α-glucosidase enzymes.	^34– 37, 39, 41– 43^
Aerial parts (stem, leaf and pods)	Ethanólic		HbA1c decrease		
Seed	Powder		Increase of plasmatic insulin		
Leaf	Aqueous and methanolic	Cardiovascular diseases	Cardioprotector	Decrease lipid peroxidation products	^38, 44, 45^
			Antihypertensive	Increase cardiac antioxidant enzymes	
Seed	Dust		Decreased heart rate	Activation of the eNOS-NO-sGC pathway	
Leaf	Ethyl acetate, hydroalcoholic, ethanolic	Renal insufficiency	Nefroprotective	Antioxidant	^46– 48^
Bark	Methanolic			Antiinflamatory	
Seed	Powder		Normalizes renal function markers		
Leaf	Powder, methanolic	Obesity	BMI decrease	Regulation of the expression of genes involved in the regulation of body weight (leptin, adipo-nectin and resistin).	^40, 49– 51^
Bark	Aqueous		Decrease in total cholesterol, triglycerides and LDL-C and increase in HDL-C.		
Leaf	Ethanolic	Arthritis	Antiarthritic	Inhibition of arthritic edema	^52^
Leaf	Ethanolic, Methanolic	Neurodegenerative diseases	Neuroprotective	Decreased activity of apoptotic brain markers	^53– 55^
Leaf Aerial parts (stem, leaf and pods)	Powder, hydroalcoholic, ethanol, ethanolic Ethanolic	Liver damage	Hepatoprotective To prevent the toxic effect of arsenic in humans and the treatment of other chronic pathologies derived from arsenic.	Antioxidant	^46, 56– 58^

Abbreviations: **HbA1c:** glycosylated hemoglobin; **eNOS-NO-sGC**: entotelial nitric oxide synthase, nitric oxide and soluble guanylyl cyclase; **BMI**: body mass index; **LDL**: low-density lipoproteins; **HDL**: high-density lipoproteins; **BChE:** pseudocholinesterase.

### 3.3 Biological activity of *Moringa oleifera* on cancer

The International Agency for Research on Cancer (IARC) states that in 2020 there were 19.3 million new cases of cancer and nearly 10 million deaths. ^59^ According to the Global Cancer Observatory (GCO), lung cancer is the most commonly diagnosed cancer in men (14.3%), followed by prostate cancer (14.1%) and colorectal cancer (10.6%). In women, the most common type of neoplasia and the one with the highest mortality rate was breast cancer (24.5%), followed by colorectal cancer (10.6%) and lung cancer (8.4%). ^59^ It is predicted that by 2030, there will be a 32% increase in the number of people diagnosed with cancer in the Americas. ^60^

Currently, the main therapies used against cancer in medical oncology are surgery, hormonal therapy, radiation, immunotherapy, and chemotherapy. Despite their widespread use, these approaches have adverse effects because they affect cells that are in constant division, such as the bone marrow, lining of the mouth and intestines, and hair follicles. The type of side effect depends on the type of medication used, amount administered, and treatment time. The use of conventional approaches such as chemotherapy has resulted in very low survival rates for patients with advanced-stage cancer. ^61^

***3.3.1 In vitro* antitumor evidence**

With the aim of elucidating the potential antitumor capacity of various extracts from *Moringa oleifera*, Cuellar *et al*. ^62^ conducted a study related to the effect of hydrolyzed extracts from the plant leaves on human colon cancer cells, HT-29 and HCT116, showing similar cytotoxic activity in both cell lines compared to untreated cells. ^62^ According to the types of extracts evaluated in the study, it was observed that the aqueous extract, the methanolic extract, and the glucosinolate-rich hydrolysate showed the highest cytotoxic effect on the HCT116 human colon cancer cell line at concentrations of 20.1%, 21%, and 21.5%, respectively, unlike the HT-29 human colon cancer cell line, in which only the aqueous and methanolic extracts showed higher cytotoxicity at concentrations of 19.8% and 22%, respectively. ^62^

To determine the possible mechanism responsible for the observed tumor cytotoxicity associated with this plant, the apoptotic and antiproliferative activity of the aqueous extract of *Moringa oleifera* leaf in human melanoma cell lines A375, A2058, and normal human fibroblasts was assessed. This biological activity was evaluated through the implementation of the WST-1 cell proliferation assay and the Annexin V-associated apoptosis assay, showing that the aqueous extract causes growth inhibition due to apoptosis events in the target lines. ^63^ However, there was little effect on the growth of normal human fibroblasts, thus revealing the specificity of this compound. ^63^

Therefore, the aqueous extract of *Moringa oleifera* can be considered an alternative therapy for the treatment of skin cancer, while keeping in mind the importance of continuing *in vitro* and *in vivo* studies that provide scientific information about the biological activity of this plant. Several approaches associated with the study of the antitumor and chemopreventive capacities of *Moringa oleifera* are summarized in Table 2.

**Table 2.** *In vitro* potential uses of *Moringa oleifera* regarding cancer.

Plant part	Biological activity	Disease	Effect	References
Leaf Whole plant Flowers	Citotoxic activity	Colorectal cancer, breast cancer,laryngeal cancer, cervical cancer, lung cancer, prostate cancer and leukemia	The presence of quinic acid, octadecanoic acid, palmitic acid (hexadecanoic acid), alpha-tocopherol (vitamin E), and β-sitosterol induce toxicity.	^62, 66– 75^
Leaf Seed Root	Apoptotic activity	Skin cancer, Cervical cancer	Cellular morphological alterations due to membrane shrinkage, nuclear contraction, condensation, and fragmentation of DNA.	^7, 63, 76– 82^
Fruit Leaf Seed	Cell viability	Liver cancer, lung cancer, breast cancer	The extract from different parts of *Moringa oleifera* shows great variability with respect to cancer cells, since the toxic effect of the extract is lower in normal cells than in cancer cells.	^64, 83, 84^
Leaf Seed	Antiproliferative activity	Lung cancer, prostate cancer, liver cancer, and squamous cell carcinoma	The leaves and seeds of *Moringa oleifera* possess antiproliferative properties as demonstrated by an increase in oxidative stress that leads to apoptosis of tumor cells.	^85– 87^
Leaf	Anticancer activity	Epidermoid cancer	Extracts from *Moringa oleifera* (n-hexane, chloroform, ethyl acetate, and methanol) were studied. The methanolic extract showed the highest effectiveness in inhibiting cancer cells.	^88^
Leaf, bark	Cell survival	Ileocecal cancer and breast cancer	Leaf extracts mainly consist of thiocyanates, hydrocarbons, and fatty acids, while bark extract is primarily composed of hydrocarbons, phenols, phthalates, carboxylic acids, and long-chain fatty acids, which can decrease cell survival.	^65^
Leaf and seed	Antioxidant activity	Breast cancer, colon cancer, liver cancer, leukemia and adenocarcinoma.	Moringa's ethanol extract exhibits antioxidant activity, which is characteristic of its high concentrations of phenols and flavonoids.	^89, 90^

Attempting to explore the potential of the Moringa plant not only regarding its leaves but also other elements, Adebayo *et al.* ^64^ evaluated the cytotoxic activity and inhibition of cell proliferation of crude extracts and fractions of MO seeds on breast adenocarcinoma cells (MCF7) and normal breast cells (MCF10A) and found an insignificant effect of the crude ethanol extract on the proliferation of MCF7 cells. ^64^ In contrast, the dichloromethane fraction of the crude ethanol extract, the hexane fraction of *Moringa oleifera* seed, and the crude aqueous extract inhibited proliferation. ^64^ The observed cell inhibition effects in this study were most likely due to the presence of phenols. The hexane fraction of the ethanol extract exhibited the best effect, as it showed antitumor activity against MCF7 cells, while having limited cytotoxicity against normal breast cells. ^64^

To determine the properties of *Moringa oleifera* in other tumor cell lines, a study targeting breast cancer cells (MDA-MB-231) and colorectal cancer cells (HCT-8) was conducted with the aim of identifying phenotypic changes, effects on cell viability, apoptosis, and alteration of the cell cycle. The results did not show a significant decrease in cell populations when exposed to different parts of the plant; however, the seed extract decreased cell motility and colony formation in both cell lines, despite not directly affecting their survival. ^65^

Regarding to the whole plant studies Diab *et al*. ^91^ conducted a research aimed to explore the *in vitro* antiproliferative potential of extracts from the entire *Moringa oleifera* plant on cell lines of lung cancer (A549), prostate (PC-3), breast (T47D and MCF-7), colon (HCT-16, Colo-205), and leukemia (THP-1, HL-60) using two methods: cytotoxicity assay and MTT assay. The results showed *in vitro* cytotoxic activity of the plant extract actively inhibiting the growth of cancer cells in the cell lines A549, PC3, MCF-7, and HCT ^92^; however, it showed a moderate effect on T47D, Colo-205, THP-1, HL-60, and K562 cells. ^91^

***3.3.2 In vivo* antitumor evidence**

In an effort to identify the potential therapeutic role of *Moringa oleifera* in preclinical trials, Barhoi *et al* published in 2021 the results of a study evaluating the cytotoxic activity of this plant in BALB/c mice with mammary adenocarcinoma. The mice were administered aqueous leaf extract for a week, and after follow-up, a reduction in tumor weight and volume was observed with increased survival of the mice, without significant alterations in the liver, kidney, or hematological parameters after the treatment. In parallel, *in vitro* evaluations were carried out, showing the dose-and time-dependent cytotoxic activity of the plant in two cell lines: HEp-2 human laryngeal carcinoma cells and EAC (Ehrlich Ascites Carcinoma) mouse mammary adenocarcinoma cells. Regarding the mechanisms involved in the observed cytotoxic effects, flow cytometry analysis confirmed the significant induction of tumor cell apoptosis by changing the mitochondrial membrane potential in the EAC cell line. ^66^

Another *in vivo* model considered the use of Sprague Dawley rats in which breast cancer was induced through treatment with 7,12-dimethylbenz(a) anthracene (DMBA), in order to analyze whether the combination of ethanolic extract of *Moringa* leaves and ethanolic extract of papaya leaves (Carica papaya L) ^93^ could delay the appearance of tumor tissue. The results showed that there were different changes in body weight among the groups of rats for 12 weeks, and the appearance of tumor tissue was detected in the 7th week in the DMBA comparison group, while in the positive control group and the combined extract group of *Moringa oleifera* and *Carica papaya* leaves, the solid period began to be detected in the tenth week. ^93^ According to histopathological tests, the growth of tumor tissue was found in all test animals, concluding that the combination of ethanolic extract of Moringa and Carica leaves at a dose of 150 mg/kg delayed the formation of cancerous tissue ^93^; therefore, they can be considered as chemopreventive agents.

Similarly, Sadek et al. evaluated *in vivo* the chemoprophylactic efficacy of *Moringa oleifera* leaf ethanolic extract against hepatocellular carcinoma in male Wistar rats (40 male Wistar rats). ^94^ After determining the appearance and histological changes in the liver, they found that rats treated with *Moringa oleifera* leaf extract (MOLEE) showed a reduction in liver enlargement as well as in the bumps and morphological alterations that this organ had compared to the group of rats treated with diethylnitrosamine (DEN), indicating that MOLEE treatment generated a favorable change in hepatic architecture. ^94^

Continuing the trend regarding the study of ethanolic extracts, in 2020, the results of the effect of MOLEE on signaling pathways in response to DNA damage to protect hepatic tissue from apoptosis triggered by Cobalt Chloride (CoCl _2_) in 40 male Sprague-Dawley rats were reported. The study showed that MOLEE reduced CoCl _2_-induced hepatic genotoxicity through the transcriptional induction of DNA damage repair genes, ^95^ thus suggesting that MOLEE is a promising candidate for the food, nutraceutical, and pharmaceutical industries. Several studies approaches associated with the study of the antitumor and chemopreventive capacity of *Moringa oleifera* are shown in Table 3.

**Table 3.** *In vivo* potential of *Moringa oleifera* regarding cancer.

Plant parts	Biological activity	Potential Uses of *Moringa oleifera* Extract in Neoplasms	Effect	References
Leaf	Cell viability	Lung and liver cancer	*Moringa oleifera* leaf extract causes a decrease in liver cell viability (HepG2) after oral administration to mice.	^86^
	Anticancer activity	Dalton's lymphoma, epidermoid cancer, breast cancer	The F1 fraction from *Moringa oleifera* leaves showed high anticancer activity when tested in in vivo mouse models.	^66, 73, 88^
	Antiproliferative activity	Breast cancer and liver cancer	The ethanolic extract of *Moringa leaves* has an antiproliferative effect on human tumor lines, this effect is largely attributed to quercetin and moringinin.	^96^
	Apoptosis	Hepatocellular carcinoma, colorectal cancer	*Moringa oleifera* leaf minimizes the protein expression of Bcl-2, Bcl-xl and B-arrestin-2.	^46, 92, 94^
	Antioxidant activity	Colorectal cancer	Moringa's bioactive compounds, such as total dietary fiber and phenolic compounds, may have chemopreventive and antioxidant capacity.	^97^
	Inhibition of tumor growth	Pancreatic cancer, prostate cancer and breast cancer	*Moringa oleifera* leaf extract decreased pancreatic cancer cell survival and significantly inhibited tumor growth.	^72, 93, 98^
	Chemoprotective activity	Hepatocellular carcinoma	*Moringa oleifera* leaf extract (MOLEE) exhibits chemoprotective activity against diethylnitrosamine (DEN)-induced hepatocellular carcinoma.	^94^
Fruit	Inhibition of tumor growth Apoptosis	Skin cancer (Melanoma) and colon cancer Colon cancer	*Moringa oleifera* fruits had effects in delaying the growth of skin tumors in mice. *Moringa oleifera* fruits (pods) possess suppressive capacity in carcinogenic processes and models in cancers such as colon cancer.	^93, 99– 101^
			*Moringa oleifera* fruit or pod, boiled and known as BbMO showed effect as a chemotherapeutic agent against colon cancer by inducing the process of apoptosis.	
Seed	Cytotoxicy	Pulmonary mucoepidermoid carcinoma, adenocarcinoma of colon and liver	*Moringa oleifera* aqueous seed extract shows no toxicity towards non-tumor cells in mice. Besides, it is an extract that has anti-inflammatory and moderate antitumor activity.	^102^
	Anticancer activity	Mama cancer	It was observed that the mixture between leaf and seed residues in different proportions exhibits anticancer potential by interfering with the signal transduction cascade responsible for cancer proliferation and progression.	^103^
Flowers	Antitumor activity	Sarcoma	The extract obtained from *Moringa oleifera* flowers showed antitumor effects at doses of 15 or 30 mg/kg, since there were reductions in tumor mass of about 97.9% compared to the negative control.	^68^

### 3.4 Biological activity of *Moringa oleifera* on infectious diseases

Antimicrobial resistance crises must be urgently managed. The Global Action Plan on Antimicrobial Resistance acknowledges and addresses the variability of resources available to countries to fight antimicrobial resistance and the development of new antimicrobials by the pharmaceutical industry. ^104^ This has led to several studies in the last decade focusing on the biological activity of phytochemicals from *Moringa oleifera* against different infectious agents. ^105– 107^

3.4.1 *In vitro* antimicrobial properties

Within the wide range of characteristics exhibited by *Moringa oleifera* for the treatment of chronic diseases, the potential of extracts from different parts of the plant as antimicrobial agents has also been elucidated. Dzotam *et al.* ^108^ evaluated the antibacterial activity of the methanolic extract of *M. oleifera* leaves against sensitive and resistant strains of *Escherichia coli, Enterobacter aerogenes, Klebsiella pneumoniae, Pseudomonas aeruginosa*, and *Providencia stuartii*, as well as their synergistic effects with some antibiotics, finding growth inhibition in 13 (68.4%) of the 19 strains used. ^108^ Interestingly, it was also observed that the *M. oleifera* extract required one of the lowest minimum inhibitory concentrations compared to the other evaluated plants to counteract the growth of a multidrug-resistant strain. ^108^ Additionally, this extract showed synergistic effects with most of the antibiotics tested. ^108^

More recently and in line with Dzotam’s work, Umamaheswari *et al*. (2020) demonstrated the antimicrobial role of the plant, specifically against methicillin-resistant *Staphylococcus aureus* (MRSA) by evaluating the antimicrobial and antibiofilm potential of extracts from four plants, including *Moringa oleifera.* The ethanolic extract obtained from M. O leaves showed the lowest minimum inhibitory concentration (MIC) (250 μg/mL) and minimum bactericidal concentration (MBC) (500 μg/mL) among the evaluated plants. ^109^ The antimicrobial potential was attributed to terpenoids and flavonoids, which are the main groups of phytoconstituents with antimicrobial properties that are naturally present in plants. Furthermore, *M. oleifera* extracts showed–30-60% inhibition of biofilm formation, and other findings revealed that the extracts were not toxic and had minimal hemolytic activity. ^109^ Table 4 summarizes some *in vitro* experimental approaches focused on demonstrating the anti-infectious capacity of different parts of the *Moringa oleifera* plant.

**Table 4.** *In vitro* potential of *Moringa oleifera* related with in infectious diseases.

Plant part	Extract fraction	Potential use	Pathogen	References
Leaf	Solvents (acetone and methanol)	Antimicrobial in co-infections associated with COVID-19	*Pseudomonas aeruginosa, Escherichia coli, Moraxella catarrhalis, Serratia spp., Enterobacter spp., Staphylococcus aureus* and *Candida tropicalis*	^112^
	Methanolic and aqueous	Antibacterial activity	Gram-positive bacteria *(Staphylococcus aureus and Streptococcus agalactiae)* y gram-negative *(Salmonella typhi and Shigella boydii)*	^113^
	Infusion		*S. aureus, K. pneumoniae, E. coli* and *P. aeruginosa*	^114^
	Methanolic	Activity against gram-negative MDR bacteria	*Escherichia coli, Enterobacter aerogenes, Klebsiella pneumoniae, Pseudomonas aeruginosa* and *Providencia stuartii*	^108^
	Ethanolic	Antimicrobial and anti-biofilm activity MRSA	Methicillin-resistant *Staphylococcus aureus*	^109^
		Anti-influenza activity	Influenza A (H9) Virus	^115^
		Anti-leishmanial properties	*Leishmania major*	^116^
	Methanolic, aqueous, and petroleum ether	Inhibition of early events of the replication cycle	HIV-1 lentiviral vector	^110^
	Aqueous	Antibiofilm and antiviral	*S. aureus*, HSV-1 and HSV-2	^117^
		Antimalaria	*Plasmodium falciparum*	^111^
	Ethanolic Aqueous Hidroalcoholic	Antibacterial and antifungal activity	*Staphylococcus aureus, Bacillus subtilis, Escherichia coli, Pseudomonas aeruginosa, Candida albicans Aspergillus niger* and *Rhizopus spp.*	^118^
			*S. aureus, P. aeruginosa* and *E. coli, C. albicans* and *A. fumigatus*	^119^
			*E. coli, K. pneumoniae, S. aureus* and *E. faecalis*	^120, 121^
Flower	Methanolic Aqueous Isolated trypsin inhibitor protein: MoFTI Aqueous	Antibacterial and antifungal activity Trypanocidal and immunomodulatory agent in Chagas disease Antibacterial activity	Gram-positive bacteria *(Monococcus luteus, Bacillus subtilis* and *Staphylococcus aureus)* and gramnegativas *(Salmonella paratyphi, Klebsiella pneumoniae* and *Pseudomonas aeruginosa*) *Cándida albicans, Aspergillus fumigat*us and *Aspergillus niger* *Trypanosoma cruzi* trypomastigotes *Klebsiella pneumoniae* and *Staphylococcus aureus*	^122– 125^
Seed	Methanolic	Antiviral	Influenza A virus	^126^
		Anthelmintic activity	*Fasciola hepatica*	^127^

Abbreviations: **MDR**, multidrug-resistant; **MRSA**, methicillin-resistant *Staphylococcus aureus*; **Mpro**, major protease of SARS-CoV-2 essential for virus replication; **HIV-1**, human immunodeficiency virus type 1; **HSV-1** and **HSV-2**, herpes simplex virus type 1 and type 2.

Regarding the antiviral potential of the plant, the activity of different leaf extracts against the infectivity of the lentiviral vector HIV-1 has been studied. It has been found that all the tested extracts, including methanol (MM), aqueous (AM), and petroleum ether (EM) extracts were active against the vector, inhibiting early events of viral replication in HeLa cells in a concentration-dependent manner, with IC _50_ values of 7.17 μg AM/mL, 7.72 μg MM/mL, and 7.59 μg EM/mL. ^110^ Another significant finding is related to the selectivity index of *M. oleifera* extracts, as they showed sufficiently high selectivity towards the lentivirus, indicating that the observed antiviral activities were not due to cytotoxicity, but rather to the presence of phytochemical components such as saponins, alkaloids, glycosides, tannins, carbohydrates, flavonoids, resins, acidic compounds, and proteins. ^106^

It has also been demonstrated that the leaves of this plant possess antiparasitic characteristics, as shown by Cudjoe *et al.,* ^111^ who studied the short-term (72 h) and long-term (7 days) effects of aqueous leaf extracts from four selected plants collected from the western region of Ghana on the growth of NF54 (chloroquine-sensitive), CamWT_C580Y (artemisinin-sensitive), and IPC 4912 (artemisinin-resistant) strains of *Plasmodium falciparum.* Long-term treatment with derivatives of *Moringa oleifera* leaves at a dose of 100 μg/mL inhibited 80% of the development of asexual forms of the NF54 strain. ^111^

Such antiparasitic potential has been revealed not only in leaves but also in flowers. The anti- *Trypanosoma cruzi* activity and cytotoxicity in mammals of the aqueous extract of *M. oleifera* flowers, as well as the trypsin inhibitor protein isolated from this part, called MoFTI, have been evaluated. It was found that both the aqueous extract and MoFTI induced lysis of *T. cruzi* trypomastigotes with median lethal concentrations at 24 hours (CL _50_/24 h) of 54.18 ± 6.62 μg/mL and 41.20 ± 4.28 μg/mL, respectively. ^124^ Both products showed low toxicity to murine peritoneal macrophages and Vero cells, with cytotoxic concentrations greater than 400 μg/mL at 50% (CC _50_). ^124^ This study postulated that the flavonoids present in the extract were responsible for the observed cytotoxicity, as suggested by other studies.

The above is consistent with the findings from a study conducted by Nova *et al*. (2018) where treatment with MoFTI for 24 hours induced the lysis of *T. cruzi* tripomastigotes and did not affect the viability of human peripheral blood mononuclear cells (PBMCs). ^123^ The effects of MoFTI on the immune response of *T. cruzi*-infected human PBMCs showed that treatment with this compound at a dose of 87 μg/mL for 48 h stimulated the release of tumor necrosis factor (TNF) α and interleukin (IL) 10 by infected PBMCs, compared to those treated with the conventional drug for the acute phase of Chagas disease, benznidazole, which had no effect on cytokine modulation in infected and uninfected cells. ^123^ Thus, MoFTI is a potential trypanocidal and immunomodulatory agent in Chagas disease that can prevent its chronicity, so it should be taken into account and considered for future formulations developed by the pharmaceutical industry.

Similarly, *Moringa oleifera* also exhibits antiviral effects in substances obtained from its seeds, as demonstrated in studies on its antiviral properties against influenza A virus (IAV) using methanol extract and apparent regulation of transcription factor EB (TFEB). Initially, *Moringa oleifera* showed low cytotoxicity towards RAW264.7, and protected infected cells from cytopathic effects (CPE), inhibiting cell lysis by up to 93.7%. ^126^ Furthermore, the hemagglutination assay was positive, indicating that the viral load in the groups treated with the extract was significantly reduced, particularly at concentrations of 5 and 10 μM. ^126^ Additionally, this extract suppressed autophagy in H1N1-infected cells, and it was established that this suppression was dependent on the decrease in TFEB levels in the nucleus of treated cells. Similar to the observations related to the MoFTI protein, the methanol extract of the seed also showed immunomodulatory properties, reflected in the reduction in TNF-α, IL-6, IL-1β, and IFN-β levels, suggesting that this extract may decrease the levels of inflammatory cytokines induced by H1N1 infection by inhibiting TFEB. ^126^

Regarding the potential antiparasitic activity of *Moringa oleifera* seeds, Kandil *et al*. ^127^ evaluated the *in vitro* and *in vivo* effects of the methanol extract of plant seeds on *Fasciola hepatica* as an alternative treatment. They found that complete inhibition of development and total death of immature *Fasciola hepatica* occurred within 48-72 hours after treatment at concentrations of 10, 25, and 50 mg/mL. ^127^ In addition, the results of *in vivo* assays showed a 20% decrease in the mean egg count per gram of feces two days after treatment in infected rabbits treated with a daily oral dose of 150 mg/kg of body weight of the extract for 3 days. On the last day of treatment, the egg count decreased by 68%, and from the seventh day onwards, no eggs were detected in the feces of the animals. ^127^ Thus, *Moringa oleifera* exhibited promising and potent activity against fascioliasis.

3.4.2 Therapeutic properties evaluated *in silico.*

Approaches focused on demonstrating the antimicrobial characteristics of Moringa in preclinical assays are very scarce ( Table 5), but interestingly, studies based on computational tools were found, which were designed to elucidate the specific role of some molecules from *M. oleifera* extracts, all of which focused on the novel SARS-CoV-2 virus. These *in silico* studies evaluated active compounds from the plant to determine their inhibitory properties against the Mpro protease of SARS-CoV-2, a key protein involved in the pathogenesis of COVID-19, finding good docking with the protein, with kaempferol showing the highest inhibitory effect against SARS-CoV-2 Mpro. Quercetin, a compound present in moringa, showed significant assembly as well, indicating that the active phytoconstituents of this plant exhibit potential relative inhibitors against SARS-CoV-2 Mpro and pharmacological characteristics similar to hydroxychloroquine, making them possible antiviral candidates in the fight against COVID-19. ^128– 130^ Other authors also mention that components such as niazirin, quercetin, moringin, ^131^ apigenin, and ellagic acid, ^132^ have an inhibitory role on the transmembrane serine protease 2 in SARS-CoV-2 infection and inhibition of two non-structural proteins 9/10 of SARS-CoV-2, respectively. Table 5 shows computational studies developed to demonstrate the anti-SARS-CoV-2 potential of *Moringa oleifera.*

**Table 5.** *In silico* approaches of *Moringa oleifera* regarding infectious diseases.

Plant part	Phytocomponent with postive docking	Potential use	Phatogen	References
Whole plant	Kaempferol and Quercetin	Inhibition of the major protease of SARS-CoV-2 (Mpro)	SARS-CoV-2	^128, 130^
	Niazirin, quercetin and moringin	Inhibition of the transmembrane serine 2 protease in SARS-CoV-2 infection.		^131^
	Apigenin and ellagic acid	Inhibition of two nonstructural 9/10 proteins of SARS-CoV-2		^133^

### 3.5 Geographical data related to the study of *Moringa oleifera* in the last decade

**Figure 2.**
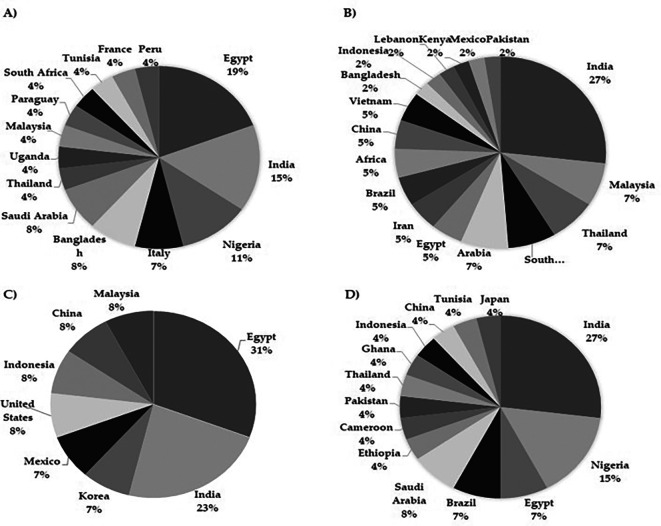
Geographical origin of publications on the biological activity of *Moringa oleifera*. A) Studies on chronic diseases B) in vitro studies related to cancer C) in vivo studies related to cancer D) Studies in infectious diseases.

The compilation of scientific material developed here has allowed not only to understand the research trends of several authors exploring the potential of *M. oleifera* in different clinical conditions but also to elucidate the geographical landscape of the sources of such publications. As expected, the majority of publications originated from Asia, since the plant is native to these regions, with India and Egypt being the two main countries where research on the biological activity of *M. oleifera* in human health is conducted, particularly in chronic diseases ( Figure 2A).

It is noteworthy that *Moringa oleifera* leaf extract (MOLE) is known for its various cytoprotective properties owing to its high levels of antioxidants, polyphenols, and flavonoids, which improve organ function by acting as regulators of damage and oxidative stress. An example of this is a study carried out in Egypt by Abou-Zeid *et al*. ^47^ and Fathy *et al*., ^57^ where the group of rats treated with MOLE showed less impact than the untreated group in terms of increased serum markers such as ALT, AST, urea, and creatinine, and decreased levels of albumin and total proteins, indicating protection against hepatic/renal toxicity.

In Egypt, the neuroprotective activity of the ethanolic leaf extract described by Muhammed *et al*. ^53^ was reflected in a significant decrease in apoptotic-cerebral marker activity, such as caspase-3 and DNA fragmentation. ^55^ This resulted in nearly normal activity of the neurotransmitter acetylcholine as well as improved mitochondrial activity of NADH dehydrogenase and ATPase after treatment with the extract. These findings indicate that *Moringa oleifera* extract has an influence on the protection of mitochondrial function in neuronal cells, thus participating in the prevention of neurodegenerative diseases such as Parkinson’s disease caused by environmental factors. ^55^

India has the highest number of studies related to *Moringa oleifera* leaves. One of the research studies was proposed by Barhoi D, Upadhaya P, Barbhuiya S, Giri A, Giri S. ^66^ In 2021, this group developed an *in vivo* study on the anticancer potential of *Moringa oleifera* against ascetic and laryngeal cancers. The methodology involved two types of biological assays: the trypan blue dye exclusion assay and the MTT assay. The MTT assay revealed that treatment with *Moringa oleifera* induced dose- and time-dependent toxicity in both cancer types. This study highlights the potential of *Moringa oleifera* leaf treatment to induce apoptosis in cancer cells, indicating that this plant could be an excellent candidate for the development of new cancer therapies.

In the context of *in vitro* antitumor screening, India again stands out as the country with the highest number of studies, contributing 26% of the overall production ( Figure 2B). In 2010, Sreelatha reported the antiproliferative and apoptotic effects of the aqueous extract of *Moringa oleifera* leaves on a human tumor cell line (KB), using two types of biological assays, the cell proliferation assay and propidium iodide (PI) staining assay. The results showed that the aqueous extract significantly inhibited the proliferation of the studied tumor cell line and induced morphological changes (such as blistering of cell membrane) after 48 h of treatment, indicating apoptosis. ^7^ Moreover, cell membrane shrinkage and loss of contact with other cells were observed. Similarly, propidium iodide staining revealed nuclear contraction, condensation, and DNA fragmentation in the cells, ^7^ providing evidence of the significant biological effects of this extract, which could be prospectively used as a therapeutic and pharmacological product for treating certain neoplasms.

Another interesting perspective in the present review involves the identification of regions from which *in vivo* research on the antitumor activity of *Moringa oleifera* has been conducted, which is not as abundant as *in vitro* approaches. In this regard, Egypt has led the production of studies in this area ( Figure 2C), specifically focusing on leaves, contributing to 31% of the total related production. This indicates that Egypt has been actively involved in the research and evaluation of the anticancer potential of *Moringa oleifera in vivo.* Representative of one of these works are the observations of Khalil (2020), related with the evaluation of the effect of MOLEE on signaling pathways in response to DNA damage to protect hepatic tissue from apoptosis triggered by CoCl2 in Sprague-Dawley rats. This study demonstrated that MOLEE reduces genotoxicity induced by CoCl2, as evidenced by the expression of DNA repair genes. ^9^

Finally, concerning the biological activity of *M. oleifera* in infectious diseases, India was once again at the top of the list of articles in this field ( Figure 2D). We highlight a study originating from that country concerning the use of extracts from certain plants to synthesize metallic nanoparticles to support the generation of new nanomaterials with more potent and/or novel biological activities. Das *et al*. ^121, 122^ by performing green synthesis of copper ^123^ and bismuth ^124^ nanoparticles from hydroalcoholic leaf extracts revealed considerable antibacterial activity against *Escherichia coli, Klebsiella pneumoniae, Staphylococcus aureus* and *Enterococcus faecalis*, and relatively stronger antifungal activity against *Aspergillus niger, Aspergillus flavus, Candida albicans* and *Candida glabrata*, especially a pro-metabolic and usable inhibitory activity against Candida spp. ^120^ These findings suggest that the synthesized copper and bismuth nanoparticles may be promising therapeutic candidates for the treatment of various bacterial and fungal infections, such as candidiasis. Owing to their encapsulation, these nanoparticles can be used in direct medical applications as antimicrobial agents. An ecological advantage of green nanoparticle synthesis is the production of non-toxic materials and the reduction of waste products compared to incinerated nanoparticles synthesized by conventional chemical-thermal methods.

### 3.6 Plan parts mentioned in studies related with the biological activity of *Moringa oleifera* in chronic diseases, cancer and infectious diseases

**Figure 3.**
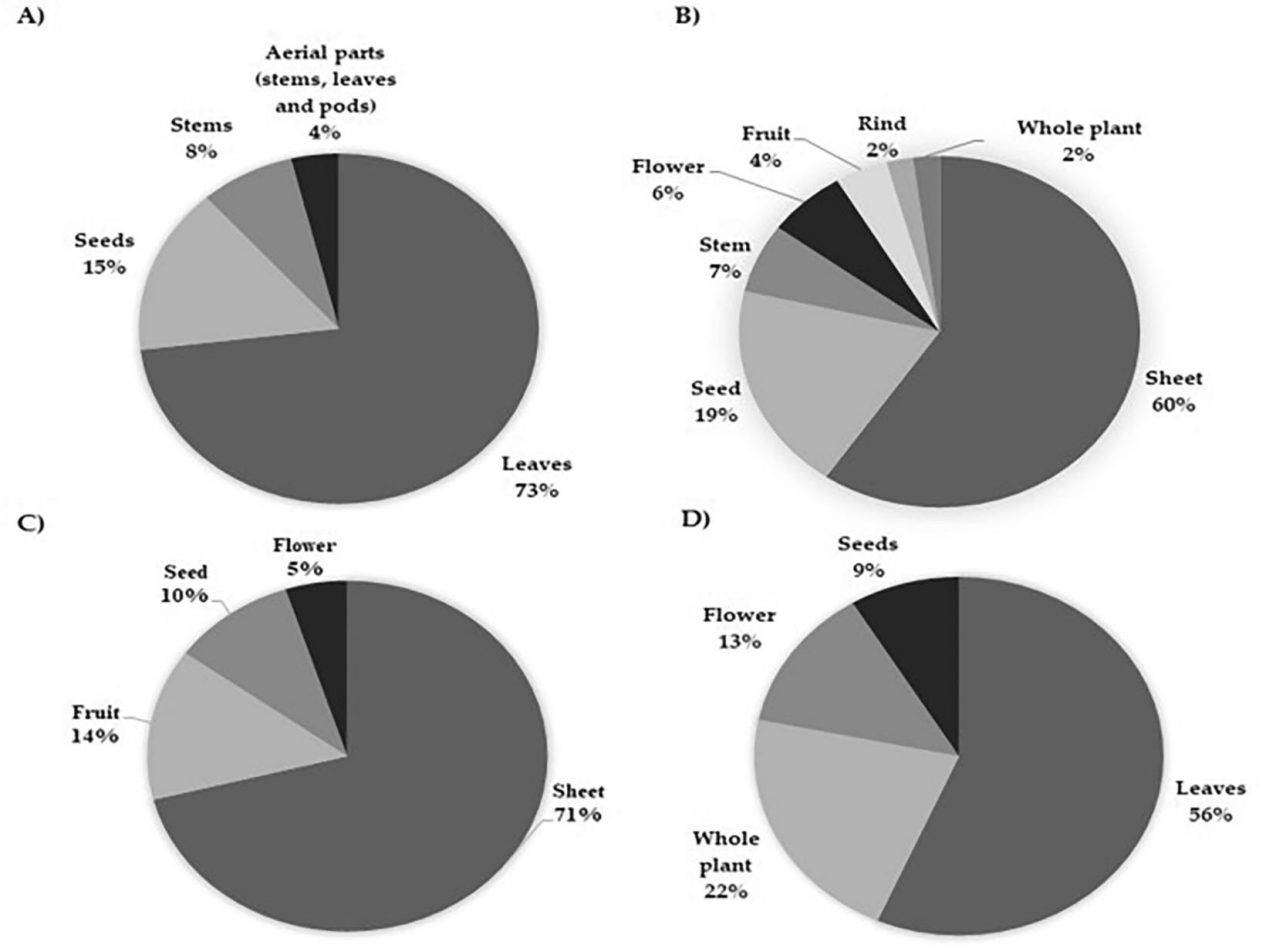
Parts of Moringa oliefera used in studies related with the biological activity in A) Chronic diseases B) in vitro cancer studies C) in vivo cancer studies. D) Infectious diseases.

Although different parts of the plant have been studied under different conditions, in all experimental and preclinical approaches, the leaf is the most extensively studied raw material, mainly because of the relative ease of obtaining a viable product for testing. However, extracts from parts other than the leaf have also revealed effectiveness in the different research contexts in which they have been tested. Studies associated with chronic diseases and *in vivo* evaluations of cancer have led to the use of the leaves of this plant, with 73% and 71% of the investigations, respectively ( Figure 3A and 3C), followed by *in vitro* assays on cancer and studies of antimicrobial potential, with 60% and 56%, respectively, that is, more than half of the investigations ( Figure 3B and 3D). From this part of the plant, most scientific production has been generated, involving different types of extracts, among which aqueous, methanolic, and ethanolic extracts stand out. A small part of the work has studied the ethyl acetate extract; among them, the research carried out by Bamagous *et al*. ^43^ showed that this extract reverses diabetes markers such as serum levels of glucose and insulin, lipid profile, and liver damage, confirming the results of a histopathological study what was evaluated at the serum level. ^43^

With regard to the study of cancer, it has already been mentioned that most studies carried out to demonstrate the antitumor role of *Moringa oleifera* contemplate the leaf as a target element. One of these *in vitro* approaches was conducted by Khalafalla *et al.* ^89^ in Egypt in 2010, where different leaf extracts were evaluated to observe the effects of the treatment on the viability of leukemia and hepatocarcinoma tumor cells using the MTT assay. The results showed that the average viability of the treated tumor cells was 25% lower than that of the control cells (human lymphoid cell line), which was 94.6%. Likewise, with regard to the viability of the HpG2 tumor cell line (liver cancer), they observed a significant decrease with respect to treatment with ethanolic extract. ^89^

Studies on the leaves of this plant have been devoted not only to revealing the ability of these extracts to attack tumor cells but also to show their harmlessness to normal cells. Thus, Suphachai in Thailand conducted research on the *in vitro* antiproliferative activity of two types of *Moringa oleifera* leaf extracts (methanol and biomethanol) on breast cancer (MCF-7 cell line), liver cancer (HepG2), and human fibroblast cells. It was found that concentrations up to 400 μg/ml of the two extracts did not induce toxicity in normal cells. ^90^ Regarding the two extracts evaluated it was also observed that biomethanol was the one that presented high antioxidant activity and potent antiproliferative activity in cancer cells. ^90^ This study reinforces evidence that *Moringa oleifera* has great potential in the field of medicine in relation to cancer chemotherapy and chemoprevention.

As observed *in vitro, in vivo* approaches also use leaves as their main raw material for research ( Figure 3C). Among these, Cuellar *et al.* ^97^ focused on determining the physicochemical and nutraceutical properties of moringa leaves and their effects in a model of colorectal carcinogenesis *in vivo.* By testing *Moringa oleifera* leaf in male mice it could be evidenced that this extract decreased the activity of harmful fecal enzymes (β-glucosidase, β-glucuronidase, tryptophanase, and urease by up to 40%, 43%, 103%, and 266%, respectively), as well as tumor incidence in male CD1 mice. ^96^ These findings suggest that bioactive compounds in moringa, such as total dietary fiber and phenolic compounds, may have chemopreventive capacity. This is the first study on the suppressive effects of this plant leaf in an *in vivo* model of colorectal carcinogenesis. ^105^

The biological activity of the plant in infectious diseases, as in the other assays, is the most studied element ( Figure 3D). Ashraf *et al*. ^115^ when comparing the cytotoxic and anti-influenza potential of ethanolic extract of *Moringa oleifera* leaves (EEOMOL) and amantadine, an anti-influenza drug, evidenced that this extract showed significantly better (P <0.05) anti-influenza activity (0.78 μg/mL to 100 ug/mL) than amantadine (12.5 ug/mL to 50 ug/mL) (112). Additionally, EEOMOL showed a higher cytotoxic concentration 50 (CC50) (100 μg/mL) than amantadine (50 μg/mL). ^115^ Based on these observations, it can be inferred that the extract could be a breakthrough in combating future influenza outbreaks, especially those caused by the influenza A (H9) virus, which has shown resistance to amantadine due to mutations in the M2 gene.

### 3.7 Extract fraction and microorganisms described in *Moringa oleifera* research

**Figure 4.**
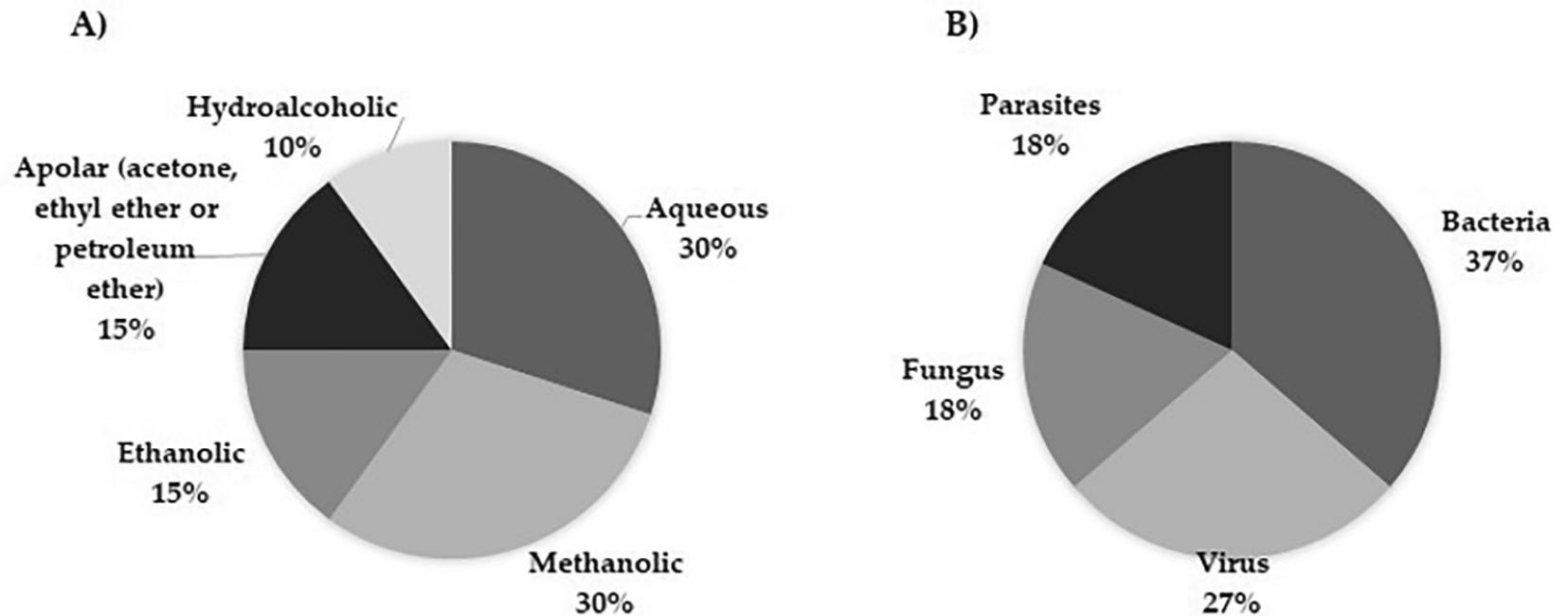
Compendium of extracts implemented regarding the biological activity of *Moringa oleifera* on infectious diseases and pathogens studied. A) Extracts B) Pathogens.

Another observation derived from this work is related to the trend that microbiological studies have shown in the past 10 years regarding the type of pathogenic agents and extracts evaluated to reveal the antimicrobial role of the plant. Thus, aqueous and methanolic extracts were used in the highest number of studies ( Figure 4A), and bacteria were the most studied microorganisms ( Figure 4B).

A noteworthy study in the line of antibacterial research was conducted by Ibrahim *et al.,* ^113^ who evaluated the antibacterial activities of methanolic and aqueous extracts of *Moringa oleifera* leaves, with and without heat treatment at 45°C, 50°C, and 55°C for 30 and 60 min, to recreate the cooking and frying conditions to which the plant is subjected when used in the diet. The non-heat-treated extracts showed the ability to inhibit the growth of gram-positive bacteria ( *Staphylococcus aureus* and *Streptococcus agalactiae*) and gram-negative bacteria ( *Salmonella typhi* and *Shigella boydii*), with the methanolic extract showing a high inhibition zone at 150 mg/mL against *S. aureus* ^113^; the aqueous extract did not show any inhibitory activity against *S. agalactiae* at a concentration of 125 mg/mL, which was attributed to the presence of low amounts of active constituents, indicating that the antibacterial activity depends on the concentration of the extract and the solvent used. ^113^

In contrast, the antibacterial activity of the heat-treated extracts was affected. The methanolic extracts maintained their activity against all tested microorganisms after exposure for 60 min at 50°C, while aqueous extracts lost their activity against *Shigella boydii* but retained it against other microorganisms. ^113^ Thus, *Moringa oleifera* could be proposed as an alternative for the control of infections caused by these four bacteria, considering that the thermal treatment of the plant reduces the antimicrobial activity of its phytoconstituents.

## 4. Conclusions and future perspectives

According to the reviewed analyses, the countries with the highest number of studies that met the inclusion and exclusion criteria were India, Malaysia, and Egypt. Regarding the review of *in vivo* cancer studies, Egypt is the country with the highest number of investigations, with 13 included articles. Similarly, in *in vitro* studies, Egypt was among the top three countries with the highest number of studies on *Moringa oleifera.* This indicates that Egypt is committed to finding new and less invasive therapies against cancer than traditional approaches. Furthermore, most studies have focused on liver cancer, which is the leading cause of cancer-related death in the country.

Breast cancer is among the most studied neoplasms in the context of this search, which has shown high incidence rates worldwide in recent years. This suggests that researchers, specifically those from Asia and Africa, have been actively seeking alternative treatments to reduce the number of associated cases and deaths. Similar trends have been observed in research conducted in India, although the articles included in this review focused on studying various types of neoplasms, including cervical cancer, which was the third leading cause of death in India in 2020.

**Figure 5.**
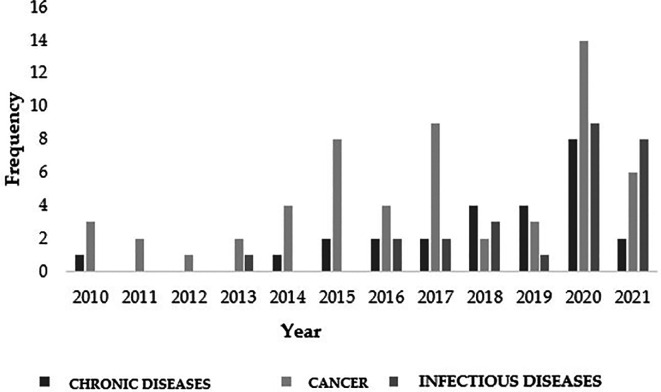
Number of worldwide *Moringa oleifera* publications for the decade from 2010 to 2020.

Indeed, in line with the study of cancer, 2020 saw the highest amount of research conducted worldwide regarding the antitumor and chemopreventive properties of *Moringa oleifera*, as indicated in Figure 5. In fact, the study of neoplasms occupied the top position in most of the temporal events that spanned the decade of the study ( Figure 5). This clear trend highlights the need for new approaches and generation of new knowledge in this field.

Regarding the study of the potential associated with different parts of the *Moringa oleifera*, and according to the findings of this complication, it was determined that the leaf is the element that apparently has the greatest biological capacity; therefore, it is the part where most research was focused, which revealed in the context of the study of cancer potential related to cell viability, cytotoxic activity, and antitumor activity in different neoplasms, than the leaf contains various components that benefit human health, which contributes to maintaining a balance between treatment and the patient’s health status. Likewise, it has come to reduce the adverse effects caused by conventional treatments, which positions it as an alternative natural treatment that is still under constant research.

Therefore, *Moringa oleifera* is a promising plant with great potential for cancer treatment, as evidenced in both *in vitro* and *in vivo* models, as described in the 60 articles analyzed in this study. This is basically due to the phytochemicals and phenolic compounds, such as quercetin and kaempferol, which allow the development of Moringa biological activities such as inhibition of cell viability through the induction of apoptosis and oxidative stress through the generation of reactive oxygen species (ROS), antiproliferative effects, and antitumor activity. In summary, this plant is a promising therapeutic alternative for cancer treatment.

Among the medicinal effects of *Moringa oleifera*, one of the most remarkable is its hypoglycemic capacity associated with counteracting one of the most relevant chronic illnesses today, diabetes mellitus, a condition that affects millions of people around the world and that does not have a treatment that achieves its definitive cure. However, there are medicines for its control, but these are not easily accessible to the entire population, and *Moringa oleifera* is widely distributed worldwide. This hypoglycemic effect is directly related to the inhibition of important enzymes, such as α-amylase and α-glucosidase, whose function is to hydrolyze carbohydrates; when these enzymes are inhibited, they delay their absorption, thus decreasing blood glucose levels. On the other hand, this plant also contributes to minimizing the sequelae of diabetes mellitus by controlling oxidative damage in the presence of multiple antioxidants and protects the target organ against damage caused by reactive oxygen species. It also contributes to counteracting the complications of the disease, such as hepatic lesions.

In addition to the evident properties of this plant related to chronic diseases, its spectrum of action is not limited to these, as its biological activity has been demonstrated against various microbiological agents. For instance, it has been found that antibacterial activity depends on the concentration of the extract and solvent used. The methanolic extract showed bacterial inhibition, whereas the aqueous extract did not. It is worth noting that antibacterial action has been observed against major pathogens, such as *Escherichia coli* and *Staphylococcus aureus*, which are highly prevalent and cause multiple diseases.

Leaves and seeds have demonstrated more effective antiviral potential, as is the case against the influenza A virus, but other viral agents have been studied. Leaf extracts exhibit potent and selective inhibition of the first steps in HIV-1 infectivity, which could serve as antiretrovirals for people suffering from HIV/AIDS, who often seek alternative and complementary therapies.

As can be seen, there is a wide range of pathologies towards which *Moringa oleifera* presents important biological activity contributing to counteracting in a similar way to the drugs traditionally used. Despite this, it is necessary to determine the pathology to be treated with a specific dose, as the concentration fluctuates according to various parameters to be controlled.

The potential use of *Moringa oleifera* is more evident because of the large number of studies described in this review. In addition, the safety and toxicity of the products derived from this plant have been intensively analyzed both *in vitro* and *in vivo*, and a considerable number of studies in humans have been conducted to demonstrate its therapeutic role in various clinical situations. However, despite all this evidence and certain intrinsic characteristics of the plant with respect to its worldwide availability and low growth requirements, the translation of the biological potential of moringa to clinical practice has not yet materialized, a fact that is evidenced by the scarcity of approved clinical trials on studies related to this plant.

While *M. oleifera* presents a wide range of therapeutic potentials, it is crucial to acknowledge the limitations that may affect its clinical application. Variability in efficacy due to environmental and genetic factors, challenges in standardizing extracts, and concerns about potential toxicity must be thoroughly investigated. For instance, discrepancies in phytochemical content based on geographic origin and cultivation conditions highlight the need for standardized cultivation and extraction protocols to ensure consistent therapeutic outcomes. Moreover, while preclinical studies offer promising results, the translation of these findings into clinical practice requires robust and well-controlled human trials to assess long-term safety and efficacy. These considerations underscore the importance of a cautious and evidence-based approach when evaluating *M. oleifera* as a therapeutic alternative.

*M. oleifera* is a plant with significant potential as both a therapeutic agent and a tool to address pressing global health challenges, including cancer and antimicrobial resistance. Unlike previous reviews, this work highlights the plant’s dual capacity to target these critical issues while integrating insights from diverse disciplines such as medicine, biotechnology, and phytotherapy. To fully realize the potential of *M. oleifera* derivatives in modern medicine, it is imperative to expand research efforts in the Western world, complementing the robust evidence already generated in regions with a long-standing tradition of studying this plant.

The therapeutic potential of *M. oleifera* is primarily attributed to its rich profile of phytochemicals and phenolic compounds, including quercetin and kaempferol. These compounds have been shown to modulate critical molecular pathways, such as the induction of apoptosis and oxidative stress through the generation of reactive oxygen species (ROS). Additionally, this review identifies novel evidence regarding its role in inhibiting cell cycle progression, enhancing the immune response, and potentiating the efficacy of conventional treatments in *in vitro* and *in vivo* models. Such findings open avenues for developing integrative and complementary therapeutic strategies targeting cancer and other diseases. ^134, 135^

Despite the promising evidence regarding *M. oleifera’s* therapeutic potential, several limitations must be addressed before its widespread clinical application. Variability in its efficacy due to environmental factors, challenges in standardizing extracts, and concerns about potential toxicity remain critical barriers. While numerous *in vitro* and *in vivo* studies demonstrate safety and effectiveness, the limited number of approved clinical trials highlights the need for more robust, well-designed research to validate these findings and ensure consistent outcomes in human populations.

This study underscores the immense therapeutic potential of *M. oleifera* as a source of innovative treatments. Future research should explore advanced strategies, such as nanotechnology, to enhance the bioavailability and efficacy of its active compounds. Moreover, integrating this plant into a bioeconomy-based business model could promote sustainable development while addressing global health challenges. By leveraging drug coating and scaffolding technologies, it may be possible to develop synergistic treatment approaches, further expanding the utility of *M. oleifera* in modern medicine.

### Ethics and consent

Ethical approval and consent were not required.

## Authors contribution

Conceptualization: A. B., B. V., K. M., and A.G.; original draft preparation: A. B, B. V, K.M.; writing: A.B,B.V,K.M, and A. G; editing, reviewing, and formatting: C.A.Z.G. and J.S: and Reviewed drafts of the paper: J.S.; Language editing and preparation of tables and/or figures: K.M., A.G.; Final approval of the review to be published. All the authors have read and agreed to the published version of the manuscript.

## Data Availability

No underlying data are associated with this article. Figshare: Data for Biological properties of Moringa oleifera: a review in last decade. https://doi.org/10.6084/m9.figshare.27233943.v3. ^132, 136, 137^ This project contains following datasets:
- dataset_1 - dataset_2 - dataset_3 - Protocol Data are available under the terms of the Creative Commons Attribution 4.0 International license (CC-BY 4.0). Figshare - PRISMA checklist for “Biological properties of Moringa oleifera: aA systematic review inof the last decade” https://doi.org/10.6084/m9.figshare.27233943.v3. ^132, 136, 137^ Data are available under the terms of the Creative Commons Attribution 4.0 International license (CC-BY 4.0).
