# Supplementary material for: Biological properties of Moringa oleifera: A systematic review of the last decade
Source: F1000Res. 2025 Jan 30;13:1390. Originally published 2024 Nov 19. [Version 2] doi: 10.12688/f1000research.157194.2 (PMC11782934; doi:10.12688/f1000research.157194.2)
Supplement: Peer review history

# Reviewer report: Reviewer response for version 2

**DOI:** 10.5256/f1000research.177200.r363555

**Published:** 2025-02-22

**Authors:** Micaela Carvajal Alcaraz

**Affiliations:** 1 CEBAS-CSIC. Campus Universitario de Espinardo, Murcia, Spain

The authors have thoroughly addressed all concerns raised in the manuscript. Specifically, they have clearly established the novelty and added value of this review by emphasizing its unique focus on the dual potential of *Moringa oleifera* in combating both cancer and antimicrobial resistance. Additionally, they have integrated data from multiple disciplines, including medicine, biotechnology, and phytotherapy, providing a broader perspective than previous reviews. The discussion has been expanded to highlight newly identified mechanisms, pathways, and potential clinical applications not extensively covered in prior literature. Furthermore, the conclusions have been revised to ensure a balanced perspective, incorporating a discussion of possible limitations such as toxicity, variability in efficacy, and challenges in standardizing extracts.

Are the rationale for, and objectives of, the Systematic Review clearly stated?

Yes

Is the statistical analysis and its interpretation appropriate?

Not applicable

If this is a Living Systematic Review, is the ‘living’ method appropriate and is the search schedule clearly defined and justified? (‘Living Systematic Review’ or a variation of this term should be included in the title.)

Not applicable

Are sufficient details of the methods and analysis provided to allow replication by others?

Yes

Are the conclusions drawn adequately supported by the results presented in the review?

Yes

Reviewer Expertise:

NA

I confirm that I have read this submission and believe that I have an appropriate level of expertise to confirm that it is of an acceptable scientific standard.

# Reviewer report: Reviewer response for version 1

**DOI:** 10.5256/f1000research.172612.r355211

**Published:** 2025-01-30

**Authors:** Pradeep Singh

**Affiliations:** 1 Debre Tabor University, Debre Tabor, Amhara, Ethiopia

**Biological properties of *Moringa oleifera*: A systematic review of the last decade**

 This review examines original research papers on *Moringa oleifera* published from 2010 to 2021. The papers focus on biological properties, especially antitumor activity and possible uses against chronic and infectious diseases. The authors gave in-depth pharmacognostical descriptions and phytochemicals from different parts of Moringa oleifera. They also talked about the plant's traditional uses, antioxidant effects, and potential to fight cancer. activity.

 The systematic review is compiled by collecting original research articles from various databases like PubMed, Scopus, and Google Scholar. The method used to put this together was very detailed. First, 2700 publications were found in databases. These were then screened to find 410 articles that met certain criteria, such as having to do with Moringa oleifera, chronic diseases, infectious diseases, biological activity, human cancer, antitumor biological activity, in vivo and in vitro activity, and anticancer properties. Finally, 129 (134 listed in reference section) publications were chosen to be included in the final draft of the systematic review.

 This compilation excludes previously published reviews, editorials, and articles published in languages other than English and Spanish.

 Suggestions

1. Abbreviations like MO, MOE, and MOSE for Moringa oleifera should be used wisely, and uniformity must be maintained throughout the article. Authors can use an abbreviation once at the beginning of the article with a full description, and then only use the abbreviation for further references.
2. The numbers of publications used for this compilation should be the same. Please check 129 publications mentioned in the abstract, 112 written in the second paragraph of the method section, and a total of 134 references listed under the reference section.
3. Authors must review English grammar and sentence construction.

 

1. As mentioned below, recheck references for duplication.

129. Soto AJ, Gomez AC, Vasquez BM, *et al.*: A Biological properties of Moringa oleifera: a review in the last decade. Publisher Full Text

 134. Soto JA, Gomez AC, Vásquez BM, *et al.*: Data for Biological properties of Moringa oleifera: a review in last decade. Dataset. *figshare.* 2024. Publisher Full Text

Are the rationale for, and objectives of, the Systematic Review clearly stated?

Yes

Is the statistical analysis and its interpretation appropriate?

Not applicable

If this is a Living Systematic Review, is the ‘living’ method appropriate and is the search schedule clearly defined and justified? (‘Living Systematic Review’ or a variation of this term should be included in the title.)

No

Are sufficient details of the methods and analysis provided to allow replication by others?

Yes

Are the conclusions drawn adequately supported by the results presented in the review?

Yes

Reviewer Expertise:

Pharmacognosy & Phytochemistry

I confirm that I have read this submission and believe that I have an appropriate level of expertise to confirm that it is of an acceptable scientific standard.

# Response

**Published:** 2025-01-30

**Authors:** C. A. Zuniga-Gonzalez

**Affiliations:** Agroecology and Agribusiness, National Autonomous University of Nicaragua, Leon. Bioeconomy and Climate Change Research Center., Leon, Leon, Nicaragua

Dear Reviewer 3,

 I sincerely appreciate your valuable feedback and observations. I have carefully reviewed your suggestions and made the necessary revisions in version 2 of the manuscript to improve its clarity and accuracy.

 First, I have standardized the use of abbreviations for Moringa oleifera (MO, MOE, and MOSE), following your recommendation to ensure consistency throughout the text. I have also corrected the discrepancy in the number of publications mentioned in the abstract, methods section, and reference list, ensuring uniformity in the data presented.

 Furthermore, I have thoroughly reviewed the English grammar and sentence structure as you suggested, to enhance the flow and readability of the manuscript. Additionally, I have checked and corrected the duplicated references you pointed out, ensuring accuracy and consistency in citation.

 Thank you once again for your attention to detail. Your feedback has been extremely helpful in improving the quality of the manuscript.

 Sincerely,

 Carlos

# Reviewer report: Reviewer response for version 1

**DOI:** 10.5256/f1000research.172612.r355204

**Published:** 2025-01-16

**Authors:** Francisco Osorio-Acosta

**Affiliations:** 1 Veracruz campus, Veracruz, Mexico

The paper has an extensive review and explained properly, however it is necessary an English redaction review. The first paragraph in introduction require connection between ideas. Some scientific names are no correctly written (italics are not used), They used a lot of abbreviations, however they don't use or are used just once (MO), furthermore, there are abbreviations that mean the same but are different (MOLE and MOLEE as an example). It is necessary to review all.

 Page 4. Figure 1 is cited in the text, however does no correspond with the information present, should be placed in other paragraph.

 Page 4, 1.3. They don't include bibliography in a paragraph.

 Page 7. 3.2 Correct Atalas, and correct redaction (The seeds of Moringa oleifera seeds have also shown potential...)

 It should be expected that authors propose better future perspectives.

Are the rationale for, and objectives of, the Systematic Review clearly stated?

Yes

Is the statistical analysis and its interpretation appropriate?

Not applicable

If this is a Living Systematic Review, is the ‘living’ method appropriate and is the search schedule clearly defined and justified? (‘Living Systematic Review’ or a variation of this term should be included in the title.)

Yes

Are sufficient details of the methods and analysis provided to allow replication by others?

Yes

Are the conclusions drawn adequately supported by the results presented in the review?

Partly

Reviewer Expertise:

Plant pathology and use of different control methods, as plant extracts.

I confirm that I have read this submission and believe that I have an appropriate level of expertise to confirm that it is of an acceptable scientific standard.

# Response

**Published:** 2025-01-18

**Authors:** C. A. Zuniga-Gonzalez

**Affiliations:** Agroecology and Agribusiness, National Autonomous University of Nicaragua, Leon. Bioeconomy and Climate Change Research Center., Leon, Leon, Nicaragua

Thank you very much for your thorough review and constructive comments. We greatly appreciate your suggestions, and we have addressed all of the points you raised. The English writing has been revised for better clarity and connection between ideas, and we have corrected the use of scientific names, ensuring proper italics are used. We have also reviewed and standardized the abbreviations throughout the paper, making sure they are consistently applied. Regarding the issues with Figure 1, it has been moved to the appropriate section. Additionally, we included the necessary bibliography in the 1.3 section and corrected the redaction in section 3.2, as you suggested. All these improvements have been made to enhance the overall quality of the manuscript.

# Reviewer report: Reviewer response for version 1

**DOI:** 10.5256/f1000research.172612.r342878

**Published:** 2024-12-14

**Authors:** Micaela Carvajal Alcaraz

**Affiliations:** 1 CEBAS-CSIC. Campus Universitario de Espinardo, Murcia, Spain

This work reviews the biological activities of *Moringa oleifera*, highlighting its antitumor, antimicrobial, and protective effects against metabolic, infectious, and chronic diseases, based on evidence from 129 studies published between 2010 and 2021. The review explores *Moringa oleifera* across various fields, including medicine and biotechnology, highlighting its versatility and addressing a wide range of bioactivities, including antioxidant, antitumor, antimicrobial, and organ-protective effects, underscoring its therapeutic potential. The reviewed findings open avenues for further exploration into drug development and integrative health strategies involving *Moringa oleifera*.

 POINTS

 2.- I have notice that there are some other reviews already published with similar subject:

- SJ Stohs, MJ Hartman. Review of the Safety and Efficacy of Moringa oleifera- Phytotherapy Research, 2015 (Ref-1) - Wiley Online Library
- KT Mahmood, T Mugal, IU Haq. Moringa oleifera: a natural gift-A review- Journal of Pharmaceutical …, 2010
- NZ Abd Rani, K Husain, E Kumolosasi. Moringa Genus: A Review of Phytochemistry and Pharmacology. - Frontiers in pharmacology, 2018 (Ref-2) - frontiersin.org
- L Gopalakrishnan, K Doriya, DS Kumar. Moringa oleifera: A review on nutritive importance and its medicinal application.- Food science and human …, 2016 (Ref-3) - Elsevier

Therefore, the novelty of this actual one should be highlight and clearly establish the added value and relevance compared to previous works.

 I suggest the following:

 -Highlight that your review specifically examines the dual potential of *Moringa oleifera i*n tackling both cancer and antimicrobial resistance—two critical and globally relevant health challenges. Also that it integrates data from diverse fields such as medicine, biotechnology, and phytotherapy, offering a broader perspective than reviews focused solely on one discipline.

 -The review should highlight any newly identified mechanisms, pathways, or potential clinical applications of *Moringa oleifera* that may not have been discussed extensively in prior reviews.

 2.- The conclusions might overstate the potential of *Moringa oleifera* without sufficient caution about its limitations, such as possible toxicity, variability in efficacy, or challenges in standardizing extracts. This fact should be implemented in discussion or conclusions.

Are the rationale for, and objectives of, the Systematic Review clearly stated?

Yes

Is the statistical analysis and its interpretation appropriate?

Not applicable

If this is a Living Systematic Review, is the ‘living’ method appropriate and is the search schedule clearly defined and justified? (‘Living Systematic Review’ or a variation of this term should be included in the title.)

Not applicable

Are sufficient details of the methods and analysis provided to allow replication by others?

Yes

Are the conclusions drawn adequately supported by the results presented in the review?

Yes

Reviewer Expertise:

Plant Sciences

I confirm that I have read this submission and believe that I have an appropriate level of expertise to confirm that it is of an acceptable scientific standard, however I have significant reservations, as outlined above.

## References

[1] : Review of the Safety and Efficacy of Moringa oleifera. *Phytother Res* .2015;29(6) : 10.1002/ptr.5325 796-804 10.1002/ptr.5325 25808883 PMC6680322

[2] : Moringa Genus: A Review of Phytochemistry and Pharmacology. *Front Pharmacol* .2018;9: 10.3389/fphar.2018.00108 108 10.3389/fphar.2018.00108 29503616 PMC5820334

[3] : Moringa oleifera: A review on nutritive importance and its medicinal application. *Food Science and Human Wellness* .2016;5(2) : 10.1016/j.fshw.2016.04.001 49-56 10.1016/j.fshw.2016.04.001

# Response

**Published:** 2025-01-18

**Authors:** C. A. Zuniga-Gonzalez

**Affiliations:** Agroecology and Agribusiness, National Autonomous University of Nicaragua, Leon. Bioeconomy and Climate Change Research Center., Leon, Leon, Nicaragua

We sincerely thank the reviewer for their insightful and constructive feedback, which has significantly contributed to improving the quality of our manuscript. Your suggestions to clearly emphasize the novelty and added value of our review compared to existing literature and to address the limitations of *Moringa oleifera* have been invaluable. These recommendations will guide us in refining our analysis and ensuring a balanced presentation of findings.
